# Structural Variations Contribute to the Genetic Etiology of Autism Spectrum Disorder and Language Impairments

**DOI:** 10.3390/ijms241713248

**Published:** 2023-08-26

**Authors:** Rohan Alibutud, Sammy Hansali, Xiaolong Cao, Anbo Zhou, Vaidhyanathan Mahaganapathy, Marco Azaro, Christine Gwin, Sherri Wilson, Steven Buyske, Christopher W. Bartlett, Judy F. Flax, Linda M. Brzustowicz, Jinchuan Xing

**Affiliations:** 1 Department of Genetics, Rutgers, The State University of New Jersey, Piscataway, NJ 08854, USA; rohanalibutud@gmail.com (R.A.); sh1436@scarletmail.rutgers.edu (S.H.); atps@outlook.com (X.C.); zhouanbo@gmail.com (A.Z.); vaidhyanathan.m@rutgers.edu (V.M.); azaro@hginj.rutgers.edu (M.A.); gwin@dls.rutgers.edu (C.G.); sherrileighwilson@gmail.com (S.W.); judyflax17@gmail.com (J.F.F.); lbrz@hginj.rutgers.edu (L.M.B.); 2 Department of Statistics, Rutgers, The State University of New Jersey, Piscataway, NJ 08854, USA; buyske@stat.rutgers.edu; 3 The Steve Cindy Rasmussen Institute for Genomic Medicine, Abigail Wexner Research Institute at Nationwide Children’s Hospital, Columbus, OH 43205, USA; christopher.bartlett@nationwidechildrens.org; 4 Department of Pediatrics, College of Medicine, The Ohio State University, Columbus, OH 43205, USA; 5 The Human Genetics Institute of New Jersey, Rutgers, The State University of New Jersey, Piscataway, NJ 08854, USA

**Keywords:** whole-genome sequencing, microarray genotyping, autism spectrum disorder, family cohort, copy number variation, structural variation, language impairment, neurodevelopmental disorder

## Abstract

Autism spectrum disorder (ASD) is a neurodevelopmental disorder characterized by restrictive interests and/or repetitive behaviors and deficits in social interaction and communication. ASD is a multifactorial disease with a complex polygenic genetic architecture. Its genetic contributing factors are not yet fully understood, especially large structural variations (SVs). In this study, we aimed to assess the contribution of SVs, including copy number variants (CNVs), insertions, deletions, duplications, and mobile element insertions, to ASD and related language impairments in the New Jersey Language and Autism Genetics Study (NJLAGS) cohort. Within the cohort, ~77% of the families contain SVs that followed expected segregation or de novo patterns and passed our filtering criteria. These SVs affected 344 brain-expressed genes and can potentially contribute to the genetic etiology of the disorders. Gene Ontology and protein–protein interaction network analysis suggested several clusters of genes in different functional categories, such as neuronal development and histone modification machinery. Genes and biological processes identified in this study contribute to the understanding of ASD and related neurodevelopment disorders.

## 1. Introduction

Autism spectrum disorder (ASD) is a neurodevelopmental disorder (NDD) that can cause difficulties in the everyday life of affected individuals, specifically in areas of social interaction, communication, and restrictive behavior. The *Diagnostic and Statistical Manual—Fifth Edition* (DSM-5) outlines the criteria for ASD diagnosis as “persistent deficits in (a) social communication and social interaction across multiple contexts, as manifested by deficits in social–emotional reciprocity, nonverbal communicative behaviors and developing, maintaining, and understanding relationships, and (b) manifested by at least two listed symptoms of restricted, repetitive patterns of behavior, interests, or activities” [1]. Studies across Asia, Europe and North America estimate that ASD affects 1–2% of the world’s population. The disorder prevails in individuals across racial, ethnic, and socioeconomic groups, and the risk of ASD is about 4.5 times higher in boys than girls [2]. ASD is variable in its presentation, and has been linked to both environmental and genetic risk factors [3,4,5]. The impact of the environment on ASD development is outside the scope of this project, and we will focus on the genetic components of the ASD etiology.

Researchers estimate the heritability of ASD varies between 50% and 90% [3,4,5,6]. Multiple twin studies showed variable concordance rates for a broad ASD phenotype in monozygotic (70–90%) and dizygotic (0–30%) twins [5,7]. Current ASD research indicates that the genetics of ASD are highly heterogeneous [8]: multiple genetic factors work in concert to produce a cumulative effect in conferring ASD susceptibility, rather than any single mutation being responsible for the ASD. For example, at least four types of rare genetic risk factors have been identified: genetic syndromes (~10% cases), chromosomal abnormalities (~5% cases), copy number variations (5–10% cases), and highly penetrant gene mutations (~5% cases) [9]. In addition, ASD is polygenic, with many common variants contributing to ASD susceptibility [10]. However, the majority of ASD cases are classified as idiopathic [11] and the underlying genetic causes are unknown, especially with regard to structural variations.

Structural variations (SVs) are large changes in the genome and contribute to many disorders [12]. Depending on the size and the discovery technology, SVs are often divided into larger copy number variations (CNVs) identified by genotyping array and smaller SVs identified by sequencing, referred to as “genomic SVs” or gSVs in the following text (the term “genomic SV” is simply a label used to describe this specific dataset for the convenience of reading). CNVs are primarily more than 10 kb in length and the genotypes are reported as the copy number of a genomic region. In contrast, gSVs are usually more than 50 bp in length and can include insertions, deletions, duplications, inversions, and mobile element insertions (MEIs). Both CNVs and gSVs are known risk factors for ASD [13,14,15,16,17]. Rare CNVs are prevalent in 5–10% of all ASD cases, and typically impact at least one but potentially several genes. There is an increased global burden for rare CNVs in ASD-affected individuals when compared to controls [18], and ASD probands inherit more rare CNVs than their unaffected siblings [16]. In particular, de novo CNVs attribute to an increased risk of ASD [19,20,21]. Furthermore, gSVs, especially de novo gSVs, also showed higher burden in ASD cases than controls and contribute to ASD genetic etiology [17]. Although gSVs are usually smaller than CNVs, they are more prevalent in populations and could have major functional impact on genes, both through direct gene disruption and affecting gene expression [22,23,24]. Compared to CNVs, the contributions of gSVs to ASD have not been studied extensively.

The New Jersey Language and Autism Genetics Study (NJLAGS) collected a cohort focused on understanding the shared genetic etiology for language impairment in ASD with more common language impairments, including specific and nonspecific language impairment disorders in a family setting [25]. Specific language impairment (SLI) is a prominent language disorder that affects approximately 7–8% of English-speaking 5-year-olds in the United States [26]. The disorder manifests with difficulties in language skills with no effect on psychology, neurology, or signs of intellectual disabilities [27]. ASD has a strong language component and genetic mapping studies showed shared genetic risks between ASD and SLI [28,29,30]. Linkage analyses of the NJLAGS cohort also identified peaks for SLI in families affected by both ASD and SLI [25,31]. Therefore, examining families with both ASD and SLI individuals could identify genes that contribute to both phenotypes and potentially explain the manifestation of SLI in some family members as opposed to ASD in others [25].

In this study, we aimed to specifically assess the contribution of SVs to ASD and SLI in the NJLAGS cohort. We analyzed SVs across the size spectrum, including CNVs identified using the genotyping array and gSVs identified using whole-genome sequencing (WGS). Across the cohort, we identified variants and genes contributing to both ASD and language impairment.

## 2. Results

### 2.1. Family Samples and Project Overview

The NJLAGS cohort recruited families that had at least one ASD patient. Many families also have additional individuals with language impairments (LI or RI). In this study, we focused on three phenotypes: ASD, LI* (the union of individuals diagnosed with either ASD or LI), and RI* (the union of individuals diagnosed with either ASD or RI) (see Methods Section 4.1 for phenotype definition details). These phenotype definitions allow us to examine the genetic variants contributing to ASD, as well as related language impairments. Microarray genotyping and WGS were conducted for 524 and 272 individuals from 109 and 73 families, respectively (Table 1). For this study, CNVs were identified using the microarray data, MEIs were identified using the WGS data, and gSVs were obtained from a previous study using the WGS data [32]. After merging the MEIs and gSVs into a gSV/MEI call set, the CNV and gSV/MEI call sets were annotated, filtered, and prioritized to obtain candidate variants and candidate genes. The final candidate genes from the two pipelines were combined into one gene set, which was then subjected to enrichment and pathway analysis.

### 2.2. Candidate CNV Identification

**Figure 1:**
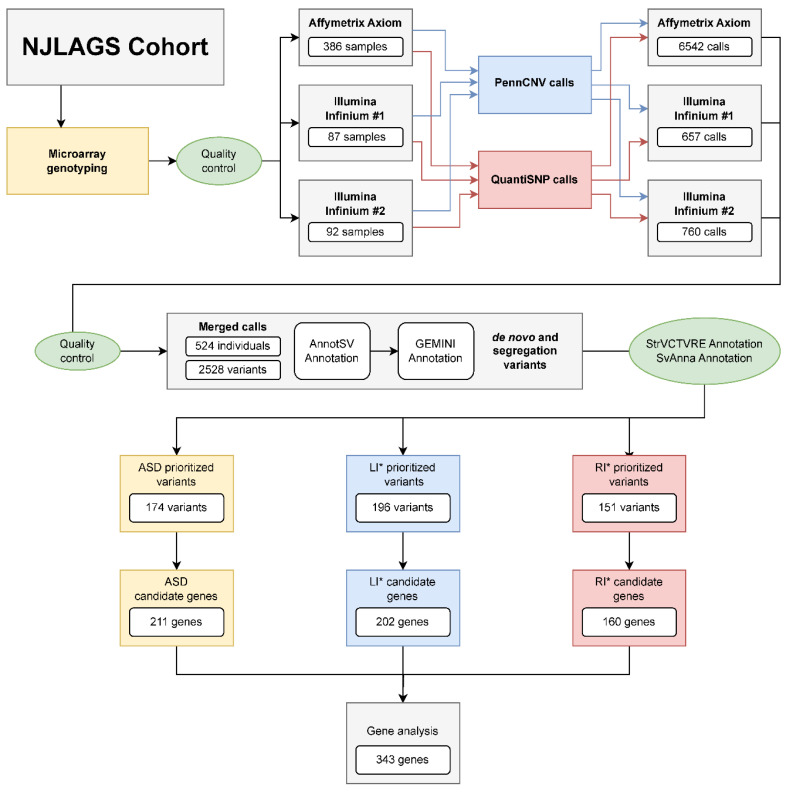
Workflow for candidate CNVs and gene identification.

First, we identified candidate CNVs and associated candidate genes. The overall workflow is outlined in Figure 1. The initial genotyping was carried out in three batches. To improve the accuracy of CNV identification, we applied two CNV calling algorithms: PennCNV and QuantiSNP (see Methods Section 4.3 for detail). After merging CNVs from PennCNV and QuantiSNP with >70% reciprocal overlap, we excluded 21 individuals who had an excess number of CNVs (>19, cohort median = 7). After QC, 524 individuals from 109 families were included in the CNV analysis. In total, 2528 CNVs passed QC and filtration (see Methods Section 4.4 for details). The sizes of CNVs range from 10 kb to 6309 kb, with a median size around 45 kb. The copy number of CNVs ranges from 0 (homozygous deletion) to 4 (homozygous duplication), with CN1 (heterozygous deletion) being the most common genotype (1641 CNVs, Figure S1A). No candidate CNV had more than four copies in any individual. ASD and unaffected individuals had the same median count of CNVs (7) as did LI* and RI* individuals (6) (Figure S1B).

Next, we prioritized the 2528 CNVs according to the absence of benign annotation, overlap with coding region, fit of an inheritance pattern, and cohort allele frequency (see Methods Section 4.5 for details). We prioritized 174 CNVs for ASD, 196 for LI*, and 151 for RI* (Figure 1, Table 2). The majority of candidates have both StrVCTVRE and SvAnna predicted pathogenicity scores >0, suggesting potential functional impact of these variants. The top candidate CNVs (StrVCTVRE score > 0.5 and SvAnna score > 1) and their associated genes are listed in Table 3. Several CNVs are known to cause congenital neurodevelopmental disorders (e.g., cerebellar atrophy, mental retardation, etc.). Finally, genes overlapping candidate CNVs were extracted and filtered on brain expression patterns. The candidate genes were later combined with candidate genes from the gSV/MEI analysis for joint analysis (see Candidate Gene Analysis Section 2.5 below).

### 2.3. A Candidate Syndromic CNV in Individual FAM23-003

**Figure 2:**
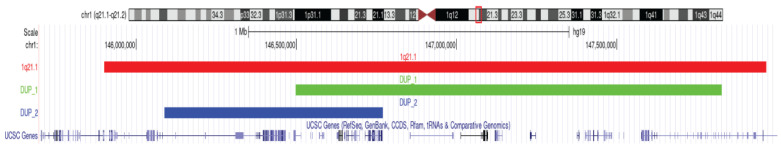
Candidate syndromic CNVs at the 1q21.1 locus. Genome Browser tracks show the 1q21.1 duplication syndrome locus (red), the duplication in the patient called by PennCNV (DUP_1, green) and the duplication called by QuantiSNP (DUP_2, blue).

We compared candidate CNVs in our patients with CNVs implicated in syndromic ASDs. One ASD patient (FAM23-003), was determined to have two CNVs overlapping the 1q21.1 duplication syndrome region, which is associated with ASD [13,14]. Both CNVs within the region are de novo heterozygous duplications (CN3): one was identified by PennCNV (chr1:146497779-147826789; DUP_1 in Figure 2) that had a >50% reciprocal overlap with the 1q21.1 locus. A second CNV was identified by QuantiSNP (chr1:146089404-146769831; DUP_2 in Figure 2), which shows partial overlap with the DUP_1. In combination these two CNVs covered 84.1% of the1q21.1 duplication syndrome locus and are likely to be one CNV that each program provided a partial definition of. Medical records for this patient indicated overlapping symptoms with the 1q21.1 duplication syndrome (see Discussion for details). Therefore, patient FAM23-003’s ASD diagnosis might be explained by this de novo CNV.

### 2.4. Candidate gSV/MEI Identification

**Figure 3:**
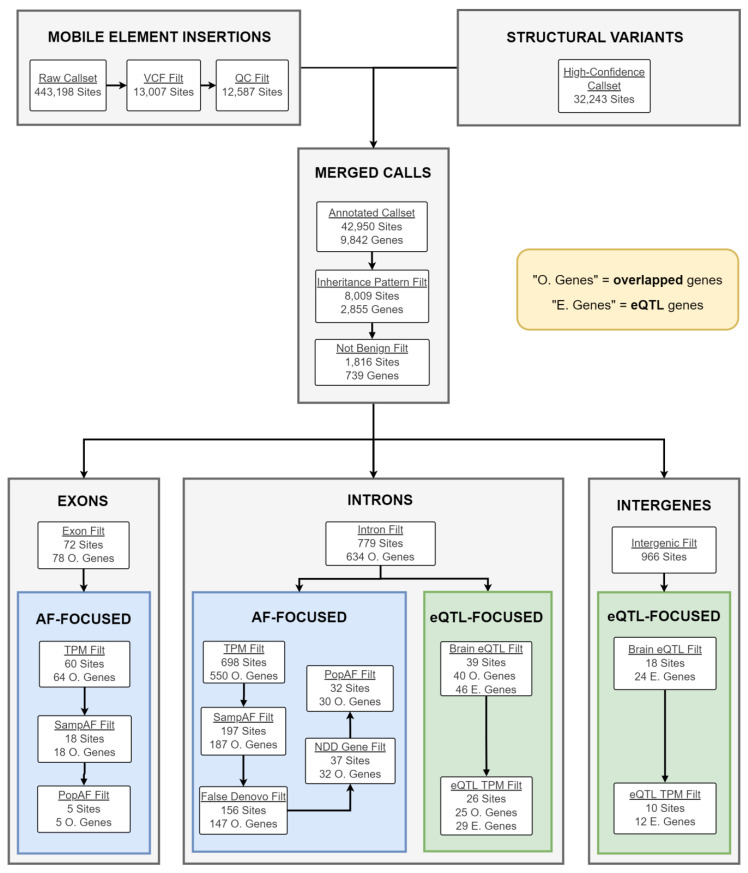
Workflow for candidate SV/MEIs and genes identification.

Next, we determined the contribution of gSVs and MEIs to the phenotypes. The overall workflow is described in Figure 3. For gSV analysis, 272 individuals from 73 families were included, including 83, 117, and 134 ASD, LI*, and RI* patients, respectively (Table 1). We identified 32,243 high-quality gSVs in the WGS data set previously [32]. Because standard gSV detection programs have low sensitivities for MEIs, we applied the program MELT to identify MEIs using the WGS data. Overall, 12,587 MEIs in 259 individuals passed QC. As expected, *Alu* is the dominant polymorphic MEIs in the cohort, followed by LINE-1 and SVA. The size distribution of the MEIs is shown in Figure S2. When combined, 42,950 gSV/MEI loci were included in the downstream analysis (see Methods Section 4.7 for QC and and Section 4.8 for merging details). After applying the inheritance pattern and known benign variant filtering, 1816 variants were identified.

To identify variants contributing to each phenotype, we applied two pipelines (Figure 3), the first of which was an “AF-focused” pipeline that is focused on rare variants within the genic regions (i.e., exonic and intronic variants). The pipeline identified five exonic variants, including two for ASD (Table S4). Because of the large number of intronic variants, we applied additional filters on the overlapping genes (see Methods Section 4.9 for details). After the filtering, 32 intronic variants in 30 genes were identified for the three phenotypes. Second, an “eQTL-focused” pipeline that identifies intronic and intergenic eQTL variants in brain tissues was applied. We identified gSVs and MEIs in our dataset that are considered causal eQTL variants in brain tissues in the GTEx project samples. A total of 26 intronic variants and 10 intergenic variants met these criteria and 29 and 12 eQTL genes were identified, respectively. The exonic variants and eQTL variants that are associated with gene expression in at least three tissues are listed in Table 4.

### 2.5. Candidate Gene Analysis

The CNV and gSV/MEI pipelines each prioritized a list of candidate genes across three phenotypes. These genes were then combined into a single gene list. Table 5 shows the counts of these genes, as well as the counts of families where the candidate genes were identified from. A total of 274 candidate genes were prioritized from the CNV data and 75 genes from the gSV/MEI data, including 344 total unique genes (Table S5). Among the candidate genes, 46 were reported previously in the SFARI database, and 13 additional genes were reported in the ADHDgene database or other NDD studies (Table S5). The majority of the families (86 of 112 families, ~77%) contained at least one candidate gene for each phenotype.

Five genes were identified in both CNV pipeline and gSV/MEI pipelines: *DIP2C*, *SH3D19*, *WWC1*, *DNAH17*, and *FN3K*. *DIP2C*, *SH3D19*, *WWC1*, and *FN3K* are candidate genes for all three phenotypes, while *DNAH17* is a candidate gene for ASD. Variants in *DIP2C* (disco interacting protein 2 homolog C) and *DNAH17* (dynein axonemal heavy chain 17) have been previously linked to ASD [33,34], and *WWC1* was identified as a risk gene for Tourette syndrome, another NDD known to be comorbid with ASD [35].

From the gSV pipeline, five genes were prioritized in the exonic AF pipeline, with two genes for ASD: *EXOC6* and *RNF213*. *EXOC6* (exocyst complex component 6) contains a LINE1 insertion that overlaps its exon 16 and meets the recessive segregation pattern for all three phenotypes. *EXOC6* was reported in the SFARI database as a strong candidate for ASD, and GO analysis links it to the axon development. *EXOC6* knockout mice demonstrate a lack of response to tactile stimuli and hyperactivity, indicating a disruption of nervous signaling. *RNF213* (ring finger protein 213) contains an *Alu* insertion that overlaps its exon 58 and meets the recessive segregation pattern for all three phenotypes. *RNF213* was reported as a candidate gene for Tourette’s syndrome [35]. Besides exonic variants, many genes identified from the intronic AF pipeline are strong candidates for ASD, such as *RIMS2* (regulating synaptic membrane exocytosis 2, RI*), and *ANK3* (ankyrin 3, RI*).

From the eQTL focused pipeline, we identified 40 candidate genes (Table S4). For example, an *Alu* insertion on chromosome 18 (chr18:73953939-73954219) was identified as a causal eQTL variant for *TSHZ1* (teashirt zinc finger homeobox 1) in the cerebellum samples in GTEx. The insertion segregates in two NJLAGS families for all three phenotypes (Table S4). *TSHZ1* is a high-confidence ASD gene in the SFARI database and it is important for motor neuron development in mouse [36].

### 2.6. GO Term and Pathway Enrichment Analysis

Using the final gene list, we performed GO term (Table S6) and pathway (Table S7) enrichment analysis. We found enrichment in several GO terms related to neuronal development, including *axon part,* which comprises 15 candidate genes, such as the gSV/MEI exonic candidate *EXOC6* (see candidate genes analysis) and a high-confidence ASD gene *ANK3*. Another candidate gene in the term is *NRSN1* (neurensin 1). *NRSN1* is identified overlapping a CNV (chr6: 24139942-24151395) in patient FAM44-005 with an RI* phenotype. *NRSN1* plays a role in neurite extension and has been identified as a risk gene for reading impairment [37]. Related terms, such as “dendrite extension,” “distal axon,” and “nervous system development,” were all likewise enriched. Another enriched GO term is “histone demethylase activity,” including genes *KDM4C, KDM4D*, and *JMJD1C*, all of which are known to contribute to susceptibility for NDDs [38,39,40]. In our cohort, *KDM4C* and *JMJD1C* were prioritized across all three phenotypes, and *KDM4D* for the RI* phenotype.

Along with the GO term analysis, we also looked for enrichment in pathways for NDD association. The most significant pathway (q = 0.0023) was the aforementioned “1q21.1 copy number variation syndrome”, discussed above in the syndromic CNV section. Likewise represented is the “complement and coagulation cascades” pathway, chiefly related to thrombin formation and blood cell development, but with some annotation in genes known to be associated with Tourette’s syndrome, epilepsy, and intellectual disability.

### 2.7. Protein–Protein Interaction Network Analysis

**Figure 4:**
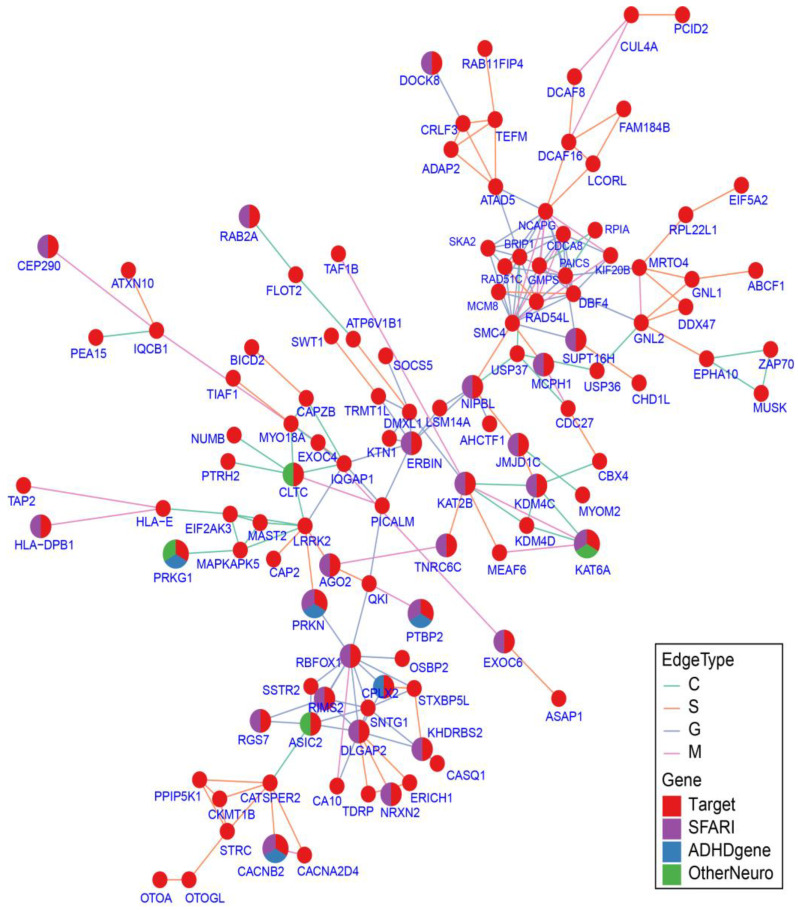
PPI Network for candidate genes. PPI network of 116 candidate genes. PPI network edges were defined by three databases: ConsensusPathDB (C), STRING (S), and GIANT_v2 (G). Edges defined by multiple databases are denoted as M. Genes that are also present in the known NDD gene list are colored accordingly (see Methods Section 4.12 for details).

Finally, we constructed a PPI network for the candidate genes to examine the shared etiology among families and phenotypes. Out of the 344 candidate genes, 116 genes connected into a single network (Figure 4, Table S8), including 29 known NDD genes.

We identified one cluster centered on hub genes *DLGAP2* (DLG associated protein 2), *RBFOX1* (RNA-binding protein, fox-1 homolog (C. elegans) 1), and *RIMS2. DLGAP2* is a known ASD risk gene associated with dysfunction of social cognition in knockout mice [41]. *RBFOX1* is a neuronal RNA-binding protein previously demonstrated to be a regulator of cytoplasmic RNA metabolism necessary for cortical development, and has also been linked to ASD [42]. *RIMS2* is also a known ASD risk gene that has been discussed in the candidate genes section above. In addition to known NDD genes, the networks include genes that were not included in our NDD gene lists but showed close association with the known genes. For example, the WNT signaling pathway genes *WNT3* and *WNT9B* have been shown to be involved in neurodevelopment and could contribute to ASD etiology [43].

The network also contains many genes involved in the histone modification process. For example, genes that overlap the GO term for “histone demethylase activity” (*KDM4C; JMJD1C; KDM4D*) form a cluster linked by *KAT2B* (lysine acetyltransferase 2B). *KAT2B* and *KAT6A* (lysine acetyltransferase 6A) are lysine acetyltransferases and have been identified as NDD genes. Specifically, *KAT6A* is specific to the RI* phenotype and has been linked to impaired speech development [44].

## 3. Discussion

ASD is a complex disorder with a highly heterogeneous genetic background. The current SFARI Gene database listed 1128 genes that have been associated with ASD. Large SVs, especially de novo CNVs and gSVs, have been consistently shown to have a higher burden in ASD cases than normal controls [13,14,15,16,17]. By systematically examining the association of different types of SVs with ASD and language impairments in the NJLAGS cohort families, we identified variants and candidate genes that are associated with ASD/language impairments.

Among the ASD patients, we identified one patient who has de novo CNVs that overlap the 1q21.1 duplication syndrome region. Duplications in this region were linked to macrocephaly/microcephaly and other NDDs, including ASD [45]. The patient with the CNVs was nonverbal and unable to take any of the diagnostic batteries. The patient also has a history of erratic moods, self-injurious behavior, and obsessive–compulsive disorder. These observations are consistent with some of the 1q21.1 duplication syndrome symptoms, including impaired communication skills. The patient does not have macrocephaly or microcephaly, another common feature of 1q21.1 duplication syndrome. A clinical test would be needed to confirm the presence of the CNV and provide a clinical diagnosis for the patient. In addition to the candidate syndromic CNVs, we identified more than 250 candidate CNVs that could contribute to the phenotypes in the cohort. Consistent with previous studies, most candidate CNVs are de novo CNVs (Table S3). Because of their large sizes and potential severe impact on affected genes, these CNVs are likely to be under strong purifying selection and will not be passed down from generation to generation.

Besides large CNVs, we also examined the contribution of other SVs, including insertion, deletion, and MEIs in the cohort using WGS data. gSVs are important contributors to ASD, although their contribution has not as been extensively studied as CNVs [17]. In addition to assessing the direct impact of gSV/MEIs at the integration sites, we also identified gSV/MEIs that are potential causal brain eQTL loci and affect nearby gene expressions. gSVs have been shown to affect nearby gene expression and typically have a bigger effect than single-nucleotide variants (SNVs) [22,23]. In our cohort, we identified 36 intronic/intergenic gSV/MEIs that passed our filtering criteria and were causal variants for brain eQTLs in the GTEx project. These variants could affect the expression of the associated brain-expressed genes in the cohort and contribute to the genetic etiology of the disorders.

Combining the candidate genes from both CNVs and gSV/MEIs, we identified 344 candidate genes, including more than 200 genes that are not included in the current database. Many new genes showed enrichment in GO terms and showed connection with known NDD genes in the PPI network. The GO enrichment and PPI network analyses pointed to several biological processes that are important for ASD etiology, such as neuronal development and histone modification. Several of the terms (e.g., “histone modification”, “actin binding”) also showed enrichment in the cohort in our previous studies of SNVs [32,46]. These genes are strong candidates for future study of their contribution to ASD.

Our study has a few limitations. One is the moderate sample size of the cohort. The family setup of the NJLAGS cohort allowed us to define and select segregating and de novo variants, which vastly reduced the number of false-positive variants. However, the comparatively small number of unaffected individuals in the sample may have hidden the signal of enrichment for CNVs in ASD probands. Furthermore, our sample was collected in two separate waves: the first with diagnoses made under DSM-IV and the second with diagnoses under DSM-5. Individuals meeting DSM-IV criteria for Asperger’s disorder would not have been considered affected in our first collection wave, having neither DSM-IV autistic disorder nor language impairment. Similar individuals would have been diagnosed with ASD under our second collection wave, due to the change in clinical diagnostic guidelines, potentially leading to a small degree of clinical heterogeneity across the two study waves. Additionally, the number of high-confidence candidate genes are limited by the sample size. Given the large number of genes involved in ASD, future study with a larger sample size could further increase the statistical power for candidate variant/gene identification.

Another limitation is that the variant discovery was based on a combination of genotyping array and short-read sequencing technologies. Using both technologies allowed us to cover most of the size spectrum and types of SVs, but neither technology is ideal for SV detection [47,48]. In particular, microarray genotyping is unable to detect inversions with neutral copy number changes. Previous studies have identified potentially causal inversions and balanced chromosomal rearrangements in certain cases of ASD [49,50]. Similar cases in our cohort would have been invisible using our current methodology. In the future, long-read sequencing technologies could provide a more complete and accurate SV profile and improve the accuracy of variant discovery [51]. Lastly, we limited our analysis to interactions among SVs. Several types of data, including coding SNVs, and microRNA variants have been identified in the NJLAGS cohort [32,46]. Integrative analysis of candidate genes from multiple types of variants could further improve the power of the analysis.

In conclusion, our multimodal analysis of SVs in the NJLAGS cohort has resulted in a list of high-confidence candidate genes that are likely to be involved in the etiology of ASD and SLI. Drawn from CNV, gSV, and MEI results, we prioritized a set of genes and biological processes that emphasize the effect of neuron growth and histone modification in ASD patients. Experimental study of the function and effect of these candidate genes could further clarify their role in the underlying mechanisms of ASD and its related phenotypes.

## 4. Materials and Methods

### 4.1. Family Selection and Phenotyping

The samples were collected by the NJLAGS [25] and the study was approved by the Institutional Review Board at Rutgers, The State University of New Jersey (IRB: 13–112Mc). NJLAGS gathered data from individuals with ASD and their family members to conduct genetic analysis. To assess their condition, each person was evaluated for ASD, oral language impairment (LI), written language impairment or reading impairment (RI), and social responsiveness during recruitment. The diagnostic process and evaluation criteria have been described in detail previously [25]. Briefly, ASD diagnoses were based on a combination of three resources: (1) the Autism Diagnostic Interview (ADI-R), (2) the Autism Diagnostic Observation Schedule (ADOS), and (3) either the *Diagnostic and Statistical Manual of Mental Disorders IV* (DSM-IV) or DSM-5, depending on date of the assessment (see [25] for details). In this study, LI is defined as either (1) receiving a score of either less than 85 on the Clinical Evaluation of Langue Fundamentals-4 (CLEF-4) test or (2) a history of language/reading difficulties with at least a score one standard deviation (SD) below their peers on at least 60% of oral language subtests. RI is defined as a score of one SD below the mean of 60% on all reading tests. Ascertainment of families in wave 1 (N = 79 families) required one person with DSM-IV autistic disorder and an additional family member with SLI, while wave 2 (N = 32 families) required one person with DSM-5 ASD and an additional family member with LI or RI [32]. To determine any shared genetic causes of ASD and LI, three phenotypes were used to define affected individuals, similar to a previous study [25]: ASD, the union of individuals diagnosed with either ASD or LI (termed LI* in the following text), and the union of individuals diagnosed with either ASD or RI (termed RI* in the following text). Because of our interest in shared genes between these conditions, the union phenotypes allow for the analysis of inherited genes that contribute to different diagnoses across generations. For example, a gene that contributes to the LI phenotype in a parent might contribute to the language phenotype in their child with ASD. These combined categories are useful for prioritizing shared genes as well as identifying de novo variants.

### 4.2. Microarray Genotyping and Quality Control

The genetic material of the NJLAGS cohort was housed and maintained at the National Institute of Mental Health (NIMH) Repository and Genomics Resource (NRGR), with the genetic and clinical data incorporated into the NRGR Autism Collection and the NIMH Data Archive (NDA). For microarray genotyping, samples were genotyped using two types of genotyping microarrays: Affymetrix Axiom 1.0 Genome-Wide CEU 1 (Affymetrix, CA, USA) and Illumina Infinium PsychArray (Illumina, CA, USA). The Illumina genotyping was performed in two batches (Table S1). The microarray genotyping was performed at Rutgers University Cell and DNA Repository (RUCDR, Piscataway, NJ, USA) following the manufacturer’s protocols. The single-nucleotide polymorphism (SNP) genotyping and quality matrix (e.g., log R ratio and B allele frequency) calculations were performed with the Axiom Analysis Suite (v4.0.3.3) and GenomeStudio Software (v2.0.4) for the Affymetrix and Illumina microarrays, respectively.

At the sample level, for the Axiom dataset, samples that had a call rate <0.95 were removed. For the PsychArray dataset, the protocol outlined in [52] for Illumina genotyping arrays was followed and all samples passed quality control (QC). At the SNP level, the Axiom samples were processed using “Best practices workflow” of Axiom Analysis Suite (v4.0.3.3) software [53], and probes that were not labeled as “best and recommended” by the software were removed. PLINK [54] was then used to filter out SNPs with <98% genotype rate (geno 0.02) or failing the Hardy–Weinberg equilibrium test at a p-value of 0.001 (hwe 0.001). The PsychArray samples were processed using the “Best practices workflow” in [52]. Probes with a GenTrain score less than 0.7 were excluded. Similarly to the Axiom samples, PLINK [54] was then used to filter out SNPs with <98% genotype rate or failing the Hardy–Weinberg equilibrium test at a p-value of 0.001.

### 4.3. CNV Identification and Quality Control

To improve the CNV identification accuracy using the microarray data, two CNV identification algorithms were used: PennCNV [55] and QuantiSNP [56]. For PennCNV, a PFB file containing the population frequency of B alleles and SNP genome coordinates was created for SNPs that passed genotyping QC. Following instructions in the PennCNV protocol, a new HMM file (Hidden Markov Model) was trained using the first 100 samples in each batch, and the resulting model file was used for CNV calling. For QuantiSNP, library files and GC files included in the program were used.

Raw CNVs were then filtered to remove CNVs that were too small to be reliably called (<10 kb), too large that they were most likely to be a chromosomal aberration or cell-line anomalies (>7.5 Mb), or called by too few probes (<5 probes). For QuantiSNP calls, CNVs with a max log Bayes factor score <10 were removed. Samples with CNV counts that were significantly greater than the rest of the samples (>median + 1.5 interquartile range) were considered outliers and removed. Sex chromosomes were excluded from the downstream analysis. Raw genotyping data and the CNV call set were deposited to the NDA under collections C1932 and C2933 and NRGR under study 39.

### 4.4. CNV Merging, Annotation, and Segregation Analysis

The following CNV analyses were carried out in a customized pipeline (https://github.com/JXing-Lab/NJLAGS_SV/tree/master/CNV_Pipeline, 20 August 2023). First, the two CNV call sets for each of the three batches were combined. The merging was performed by the custom script CNV_Builder.py. Positions were considered overlapping and merged if they shared at least a 70% reciprocal overlap with each other, with their outermost breakpoints used to define the region. Nonreciprocal overlaps, such as one variant being entirely encompassed by another but accounting for <70% of the second variant, were considered separate and were not merged. The merged CNV calls were converted into a standard variant call format (VCF) file.

CNV calls in VCF format were then annotated with AnnotSV (V. 3.0.9) [57] using the default parameters. Candidate CNVs were also compared to known ASD-related syndromic CNVs from SFARI (SFARI CNV module, https://gene.sfari.org/database/cnv/, access on 19 May 2023). The comparison was performed using *bedtools intersect* requiring a 50% reciprocal overlap [58]. The results were visualized using UCSC Genome Browser custom tracks.

To annotate CNVs for their inheritance patterns, the GEMINI (GEnome MINIng) framework was used. GEMINI is an SQL-based framework that allows querying allele information, including allele segregation in families [59]. Because GEMINI was not designed to work with CNVs, a Python program (Geminesque_2023.py) was written to apply segregation logic similar to GEMINI. The segregation pattern for each CNV was then annotated and filtered using Geminesque_2023.py and CNVs were assigned to the following inheritance patterns: de novo, autosomal recessive, and autosomal dominant. Criteria were similar to the “strict” setting in GEMINI: for de novo, parents cannot be affected, the affected child must be heterozygous for the variant, and no unaffected individuals can have the alternative allele. For autosomal recessive, all known parents must be unaffected and heterozygous for the alternative allele, while the affected children must be homozygous for the alternative allele. For autosomal dominant, affected children must have at least one affected parent and unaffected individuals cannot be homozygous or heterozygous for the alternative allele.

### 4.5. CNV Filtration and Prioritization

Subsequent steps in the filtration and prioritization processes were conducted using a custom Python script—CNV_Prioritizer.py. Candidate CNVs were filtered according to the following criteria: present in at least one affected individual; overlap with protein-coding regions; and segregated with a defined inheritance pattern. Variants with AnnotSV annotation for the benign variants (i.e., annotations in “B_gain_source,” “B_loss_source,” “B_ins_source,” or “B_inv_source”) were filtered out. For CNVs identified as a de novo variant, variants present in more than two individuals in the cohort were filtered out.

Given the neurological basis of ASD and SLI, candidate genes were filtered based on expression in brain tissues. A candidate CNV needed to contain at least one gene that is expressed in brain tissue with >5 TPM (transcripts per million). The gene expression in brain tissues was obtained from three databases: Gene Tissue Expression Project (GTEx) [60,61], the BrainSpan Atlas of the Developing Human Brain [62], and the Human Developmental Biology Resource (HDBR) [63].

### 4.6. Whole-Genome Sequencing Data and the gSV Call Set

WGS was performed on 272 individuals across 73 families in four batches by three vendors (Table S1), as described previously [32]. The gSV call set was generated using MetaSV [64]. For details of sequencing and gSV identification, see [32]. Raw sequencing data and the gSV call set were deposited to the NDA under collections C1932 and C2933 and NRGR under study 39.

### 4.7. Mobile Element Insertion Identification and Filtering

Three types of MEIs—*Alu*, LINE1, and SVA—were identified from the WGS data using MELT (V2.1.5) [65], as described previously [22]. MEIs that are present in the sequenced individual but not the reference genome are defined as “MEI Insertions.” MEIs that are present in the reference genome but not the individual are defined as “MEI Deletions.” The “MELT-SPLIT” and the “MELT-Deletion” modes were used under default settings to identify MEI Insertions and Deletions, respectively. The six MEI VCF files (three insertions, three deletions) were concatenated into one composite VCF using bcftools [66].

A series of filters were then applied to the combined MEI call set to reduce false positives. Thirteen individuals from the Knome sequencing batch (Table S1) had high no-call rates (average 43.8%) compared to the rest of the batches (average 0.09%), indicating poor variant calling quality. Therefore, these individuals were removed from the dataset. Next, loci with a missing rate >5% or a Hardy–Weinberg equilibrium test *p*-value < 1 × 10^−20^ were removed. MEI Insertions were further filtered for loci with MELT ASSESS score ≥3, VCF “FILTER” column with “PASS” or “rSD,” and split reads >2. CrossMap (v. 0.2.7) [67] was then used to lift over the genomic coordinates of the loci from the human reference genome version GRCh38 to GRCh37/hg19.

### 4.8. Merging gSV and MEI Call Sets

The following gSV and MEI analyses were carried out in a customized pipeline (https://github.com/JXing-Lab/NJLAGS_SV/tree/master/gSV_Pipeline, 20 August 2023). SURVIVOR [68] was used to merge the gSV and the MEI call sets in two steps. First, gSVs and MEIs from the same individual were merged using the following parameters: (1) breakpoint distance ≤ 100 bp, (2) supported by at least one caller, (3) agree on SV types, (4) agree on strand, and (5) no minimum length requirement. Once one gSV/MEI file was generated for each individual, all individual gSV/MEI files were merged using the same parameters to generate a combined gSV/MEI call set. This combined call set will be referred to as “gSV/MEI call set” in the following text.

### 4.9. gSV/MEI Annotation, Segregation Analysis, and Filtering

AnnotSV [57] was used to annotate the merged gSV/MEI call set using the default parameters. Variants located in known benign regions of the genome, as defined by AnnotSV, were filtered out. GEMINI v0.20.1 [59] was used to identify the inheritance patterns of gSVs, including autosomal recessive, autosomal dominant, de novo, X-linked recessive, X-linked dominant, and X-linked de novo. Variants that met any of these segregation patterns were included in the candidate prioritization. 

For genes overlapping the candidate variants, brain expression data were obtained as described in the CNV section. Brain expression quantitative trait loci (eQTL) data for gSVs and MEIs were obtained from studies of GTEx samples [22,23]. For SVs, a brain eQTL variant was defined using the following criteria: “lead_variant_type” = “SV”; “tissue” = “Brain_*”; “lead_sv_caviar_prob” > 0.1; “lead_sv_caviar_rank” ≤ 5. For MEIs, a brain eQTL variant was defined using the following criteria: “tissue” = “Brain_*”; “eqtl_origin” = “MEI”; “tissue_count_ME_isCausal” > 0.

Population allele frequency (AF) data was obtained from two sources. For MEIs, AF were extracted from the previous study of GTEx samples [22], allowing a max of 100 bp distance between the GTEx MEIs and our MEIs. For all variants that had no match in the GTEx MEI data set, the gnomAD SVs 2.1 database [69] was used to extract population AF, allowing a max of 100 bp distance between the gnomAD variants and our gSV/MEIs. A list of known NDD genes was obtained from a previous study [32] and SFARI Gene (https://gene.sfari.org//wp-content/themes/sfari-gene/utilities/download-csv.php?api-endpoint=genes, accessed on 19 May 2023) (Table S2). Gene biotype annotations were retrieved from GENCODE (v14).

### 4.10. gSV/MEI Candidate Prioritization

Filtered variants were partitioned based on their locations in the genome: exonic, intronic, and intergenic using the AnnotSV annotation. Intergenic variants were identified as variants with a null value in their “Gene_name” field. Intronic variants were identified as variants that had an annotated gene name and satisfied two criteria: (1) the “Location” field was not null, and (2) the start and end locations were within the same intron. Exonic variants were identified as variants that had an annotated gene name and (1) “Location” fields not null and (2) start and end locations in an exon or across multiple introns/exons.

Based on the variant location, two types of candidate prioritization pipelines were performed: AF-focused and eQTL-focused. AF-focused filtering identifies rare variants (in our sample and in the general population) that affect the gene they are located in. eQTL-focused filtering finds variants that are known to have a causal impact on a gene expressed in brain tissues (i.e., a brain eQTL variant in GTEx samples).

Exonic variants are likely to disrupt the function of the overlapped gene; therefore; the AF-focused pipeline was applied. Intergenic variants do not overlap any gene; so only the eQTL-focused pipeline was applied. For intronic variants, both AF and eQTL-focused pipelines were applied.

The AF-focused prioritization pipeline applied three filters: (1) TPM, (2) Sample AF, and (3) Population AF. For the TPM filter, the expression levels of a gene in brain tissues were obtained from three brain expression databases as described in the CNV section. A gene is considered expressed if any of the three TPM values was >5. Variants overlapping only genes that are not expressed in brain tissues were excluded. For the “Sample AF” filter, variants whose sample AF is higher than 5% were excluded. Similarly, the “Population AF” filter excluded variants whose population AF was higher than 5%. Due to the large number of intronic AF-focused candidates, two additional filters were applied: False DeNovo and NDD. The “False DeNovo” filter excluded variants that had a de novo inheritance pattern across multiple affected individuals. Under the “NDD” filtering, variants overlapped genes that were not in a known NDD gene list (Table S2 in [70] and SFARI Gene) were excluded.

The eQTL-focused prioritization pipeline applies two filters: (1) Brain eQTL, and (2) Brain eQTL gene. The “Brain eQTL” filter selects variants that are considered brain eQTL variants in GTEx samples (see “Brain eQTL” section above for detail). The “Brain eQTL gene” filter removes variants whose eQTL gene is not expressed in brain tissues, as defined in the TPM filter above.

### 4.11. Pathogenicity Prediction

Two pathogenicity predictors were used to determine the potential pathogenicity of the candidate variants: StrVCTVRE [71] and SvAnna [72]. StrVCTVRE (Structural Variant Classifier Trained on Variants Rare and Exonic) is a random-forest classifier designed to distinguish between benign and pathogenic SVs [71]. SvAnna is a pathogenicity predictor built off human phenotype ontology (HPO) terms and provides a pathogenicity of structural variation (pSV) score based on sequence deleteriousness and phenotype similarity [72]. Both StrVCTVRE and SvAnna were run with default parameters.

### 4.12. Gene Ontology and Pathway Enrichment Analyses, Protein–Protein Interaction Network Analysis

Gene Ontology (GO) and pathway enrichment analyses were performed using ConsensusPathDB [73]. GO terms and pathways with a false-discovery rate <0.1 were considered enriched and GO terms with <500 total genes within the group were selected to increase the specificity of the enrichment results.

Protein–protein interaction (PPI) networks were built for the final candidate genes using Python package NetWorkX [74]. ConsensusPathDB [73], STRING [75], and GIANT_v2 [76,77] were used to generate a list of known gene interactions. Detailed data processing procedures have been described previously [46,70].

## Figures and Tables

**Table 1:** Summary of families and patients.

	Phenotype	Patients	Male	Female	Families	Dominant	Recessive/De Novo
**CNV**	**ASD**	147	119	28	109	0	109
	LI*	196	146	50	109	7	109
	RI*	235	160	75	109	8	108
	**Cohort**	524	300	224	109	-	-
**gSV/MEI**	**ASD**	83	65	18	73	0	73
	LI*	117	86	31	73	7	73
	RI*	134	96	38	73	8	73
	**Cohort**	272	166	106	73	-	-

Patients, male, female: the number of affected individuals and their sexes. Families: the number of families represented by affected individuals. Dominant, recessive/de novo: the number of families whose phenotype matches a particular segregation pattern. Families with multiple trios can fall within multiple segregation categories, so the sum of the last two columns may be greater than the count of total families.

**Table 2:** Summary of candidate CNVs for the three phenotypes.

Phenotype	Prioritized CNVs	Median Length (bp)	Deletion	Duplication	StrVCTVRE >0	SvAnna >0
ASD	174	58,649	111	63	135	114
LI*	196	54,899	139	57	152	130
RI*	151	64,567	106	45	102	96

**Table 3:** Top candidate CNVs and associated genes.

Chr	Start	End	Genotype	Family	Genes	Known Phenotype (OMIM)	StrVCTVRE	pSV
1	19,540,528	19,988,894	CN1	FAM96	*AKR7A2|AKR7A3|AKR7L|CAPZB|EMC1|EMC1-AS1|LOC100506730|LOC105378614|MICOS10|MICOS10-NBL1|MRTO4|NBL1|RPS14P3|SLC66A1*	Cerebellar atrophy, visual impairment, and psychomotor retardation	0.707	9
1	19,545,053	19,596,156	CN1	FAM96	*AKR7L|EMC1|EMC1-AS1|MRTO4*		0.673	2
1	19,622,270	19,715,311	CN0	FAM96	*AKR7A2|CAPZB|SLC66A1*		0.699	3
1	19,733,320	19,988,894	CN1	FAM96	*CAPZB|LOC105378614|MICOS10|MICOS10-NBL1|NBL1|RPS14P3*		0.627	4
1	46,721,155	46,782,409	CN1	FAM19	*LRRC41|RAD54L|UQCRH*		0.752	2
1	241,829,586	241,843,408	CN1	FAM5	*WDR64*		0.594	2
2	98,317,685	98,391,355	CN1	FAM88	*C2orf92|TMEM131|ZAP70*	Autoimmune disease, multisystem, infantile-onset, 2; Immunodeficiency 48	0.627	2
6	30,337,738	30,541,852	CN1	FAM2	*ABCF1|GNL1|HLA-E|LINC02569|PRR3*		0.745	4
7	87,436,780	87,514,832	CN1	FAM138	*DBF4|RUNDC3B|SLC25A40*		0.652	3
8	176,818	2518,930	CN1	FAM30	*ARHGEF10|CLN8|DLGAP2|DLGAP2-AS1|ERICH1|FAM87A|FBXO25|KBTBD11|KBTBD11-OT1|LOC101927752|LOC101927815|LOC101928058|LOC105377777|LOC286083|LOC401442|MIR3674|MIR596|MIR7160|MYOM2|RPL23AP53|TDRP|ZNF596*	Ceroid lipofuscinosis, neuronal, 8; Ceroid lipofuscinosis, neuronal, 8, Northern epilepsy variant	0.732	7
11	94,681,989	94,732,407	CN1	FAM44	*CWC15|KDM4D*		0.694	2
15	90794,757	90,950,358	CN3	FAM27	*CIB1|GABARAPL3|IQGAP1|NGRN|TTLL13P|ZNF774*		0.561	1.2
17	27,187,789	27,434,490	CN3	FAM21	*DHRS13|ERAL1|FLOT2|LOC101927018|MIR144|MIR451A|MIR451B|MIR4732|MYO18A|PHF12|PIPOX|SEZ6|TIAF1*		0.725	1.6
17	29,107,588	29,262,773	CN3	FAM21	*ADAP2|ATAD5|CRLF3|SUZ12P1|TEFM*		0.529	1.2
17	56,584,205	57,229,716	CN3	FAM21	*MIR301A|MIR454|MTMR4|PPM1E|RAD51C|SEPTIN4|SEPTIN4-AS1|SKA2|TEX14|TRIM37*		0.592	1.4
17	56,717,956	57,017,420	CN1	FAM2	*PPM1E|RAD51C|TEX14*	Fanconi anemia, complementation group O; Breast-ovarian cancer, familial, susceptibility to, 3	0.581	2
17	57,567,551	57,875,554	CN1	FAM104	*CLTC|DHX40|LINC01476|PTRH2|VMP1*	Infantile-onset multisystem neurologic, endocrine, and pancreatic disease; Mental retardation, AD 56,	0.813	2
22	31,190,639	31,375,585	CN1	FAM27	*LOC107985544|MORC2|MORC2-AS1|OSBP2|TUG1*	Charcot–Marie–Tooth disease, axonal, type 2Z	0.742	2

**Table 4:** Candidate exonic and top eQTL gSV/MEIs and their associated genes.

Chr	Start	End	Type	Gene	Sample AF	Pop AF	Tissue Count	Pipeline
10	94,707,842	94,708,290	INS	*EXOC6*	2.32%	1.14%	-	ExonicAF
17	78,356,535	78,356,813	INS	*RNF213*	4.44%	4.49%	-	ExonicAF
22	36,561,302	36,561,477	INS	*APOL3*	1.16%	3.08%	-	ExonicAF
3	155,656,737	155,656,738	INS	*GMPS*	0.39%	0.04%	-	ExonicAF
8	66,927,128	66,927,399	INS	*DNAJC5B*	0.39%	0.26%	-	ExonicAF
10	104,645,257	104,645,587	DEL	*AS3MT*	42.88%	-	14	IntronicEQTL
4	152,340,722	152,341,553	DEL	*SH3D19;FAM160A1*	51.68%	37.30%	6	IntronicEQTL
6	170,038,129	170,038,446	DEL	*WDR27*	23.78%	26.38%	7	IntronicEQTL
6	170,044,824	170,045,154	DEL	*WDR27*	30.41%	-	3	IntronicEQTL
14	92,619,420	92,620,656	INS	*CPSF2*	14.67%	20.65%	15	IntronicEQTL
17	76,052,946	76,053,029	INS	*DNAH17*	9.07%	-	3	IntronicEQTL
20	1,546,228	1,546,508	INS	*SIRPB2*	32.82%	40.56%	5	IntronicEQTL
20	34,458,566	34,458,843	INS	*FER1L4;CPNE1*	8.30%	4.21%	6	IntronicEQTL
5	167,619	167,899	INS	*CCDC127;LRRC14B*	9.65%	4.23%	3	IntronicEQTL
6	110,102,982	110,103,230	INS	*MICAL1;AK9*	61.00%	26.96%	4	IntronicEQTL
6	116,898,686	116,898,965	INS	*RSPH4A;KPNA5*	17.95%	17.27%	13	IntronicEQTL
8	61,526,797	61,527,076	INS	*RAB2A*	25.68%	20.85%	6	IntronicEQTL
4	120,244,573	120,244,904	DEL	*C4orf3*	24.06%	-	4	IntergenicEQTL
10	124,736,004	124,736,285	INS	*PSTK*	63.71%	42.99%	3	IntergenicEQTL
4	152,732,823	152,738,842	INS	*FAM160A1*	10.81%	2.90%	3	IntergenicEQTL
9	95,680,508	95,680,786	INS	*ZNF484;BICD2;CENPP*	14.67%	9.60%	4	IntergenicEQTL

**Table 5:** Summary of genes prioritized from both CNV and SV data sets.

Phenotype	CNV		SV/MEI		Total (Unique)	
	Genes	Families	Genes	Families	Genes	Families
**ASD**	212	50	49	51	258	77
LI*	203	54	50	41	252	76
RI*	161	43	57	48	217	73
**Total**	274	62	75	58	344	86

## Data Availability

The raw sequencing reads, variants, and genotypes for all samples are available in the National Institute of Mental Health Data Archive (NDA) under collections C1932 and C2933 and NRGR under study 39.

## Supplementary Materials

The following supporting information can be downloaded at https://www.mdpi.com/article/10.3390/ijms241713248/s1.

## References

[1] American Psychiatric Association (2013). Diagnostic and Statistical Manual of Mental Disorders, (DSM-5).

[2] Christensen D.L., Baio J., Van Naarden Braun K., Bilder D., Charles J., Constantino J.N., Daniels J., Durkin M.S., Fitzgerald R.T., Kurzius-Spencer M. (2016). Prevalence and Characteristics of Autism Spectrum Disorder Among Children Aged 8 Years–Autism and Developmental Disabilities Monitoring Network, 11 Sites, United States, 2012. MMWR Surveill. Summ..

[3] Bai D., Yip B.H.K., Windham G.C., Sourander A., Francis R., Yoffe R., Glasson E., Mahjani B., Suominen A., Leonard H. (2019). Association of Genetic and Environmental Factors With Autism in a 5-Country Cohort. JAMA Psychiatry.

[4] Sandin S., Lichtenstein P., Kuja-Halkola R., Hultman C., Larsson H., Reichenberg A. (2017). The Heritability of Autism Spectrum Disorder. JAMA.

[5] Tick B., Bolton P., Happe F., Rutter M., Rijsdijk F. (2016). Heritability of autism spectrum disorders: A meta-analysis of twin studies. J. Child. Psychol. Psychiatry.

[6] Sandin S., Lichtenstein P., Kuja-Halkola R., Larsson H., Hultman C.M., Reichenberg A. (2014). The familial risk of autism. JAMA.

[7] Rosenberg R.E., Law J.K., Yenokyan G., McGready J., Kaufmann W.E., Law P.A. (2009). Characteristics and concordance of autism spectrum disorders among 277 twin pairs. Arch. Pediatr. Adolesc. Med..

[8] Iakoucheva L.M., Muotri A.R., Sebat J. (2019). Getting to the Cores of Autism. Cell.

[9] Devlin B., Scherer S.W. (2012). Genetic architecture in autism spectrum disorder. Curr. Opin. Genet. Dev..

[10] Grove J., Ripke S., Als T.D., Mattheisen M., Walters R.K., Won H., Pallesen J., Agerbo E., Andreassen O.A., Anney R. (2019). Identification of common genetic risk variants for autism spectrum disorder. Nat. Genet..

[11] Muhle R., Trentacoste S.V., Rapin I. (2004). The genetics of autism. Pediatrics.

[12] Abel H.J., Larson D.E., Regier A.A., Chiang C., Das I., Kanchi K.L., Layer R.M., Neale B.M., Salerno W.J., Reeves C. (2020). Mapping and characterization of structural variation in 17,795 human genomes. Nature.

[13] Pinto D., Marshall C., Feuk L., Scherer S.W. (2007). Copy-number variation in control population cohorts. Hum. Mol. Genet..

[14] Cook E.H., Scherer S.W. (2008). Copy-number variations associated with neuropsychiatric conditions. Nature.

[15] Stefansson H., Meyer-Lindenberg A., Steinberg S., Magnusdottir B., Morgen K., Arnarsdottir S., Bjornsdottir G., Walters G.B., Jonsdottir G.A., Doyle O.M. (2014). CNVs conferring risk of autism or schizophrenia affect cognition in controls. Nature.

[16] Krumm N., Turner T.N., Baker C., Vives L., Mohajeri K., Witherspoon K., Raja A., Coe B.P., Stessman H.A., He Z.X. (2015). Excess of rare, inherited truncating mutations in autism. Nat. Genet..

[17] Brandler W.M., Antaki D., Gujral M., Noor A., Rosanio G., Chapman T.R., Barrera D.J., Lin G.N., Malhotra D., Watts A.C. (2016). Frequency and Complexity of De Novo Structural Mutation in Autism. Am. J. Hum. Genet..

[18] Chen C.H., Chen H.I., Chien W.H., Li L.H., Wu Y.Y., Chiu Y.N., Tsai W.C., Gau S.S. (2017). High resolution analysis of rare copy number variants in patients with autism spectrum disorder from Taiwan. Sci. Rep..

[19] Krumm N., O’Roak B.J., Karakoc E., Mohajeri K., Nelson B., Vives L., Jacquemont S., Munson J., Bernier R., Eichler E.E. (2013). Transmission disequilibrium of small CNVs in simplex autism. Am. J. Hum. Genet..

[20] Sebat J., Lakshmi B., Malhotra D., Troge J., Lese-Martin C., Walsh T., Yamrom B., Yoon S., Krasnitz A., Kendall J. (2007). Strong association of de novo copy number mutations with autism. Science.

[21] Sanders S.J., He X., Willsey A.J., Ercan-Sencicek A.G., Samocha K.E., Cicek A.E., Murtha M.T., Bal V.H., Bishop S.L., Dong S. (2015). Insights into Autism Spectrum Disorder Genomic Architecture and Biology from 71 Risk Loci. Neuron.

[22] Cao X., Zhang Y., Payer L.M., Lords H., Steranka J.P., Burns K.H., Xing J. (2020). Polymorphic mobile element insertions contribute to gene expression and alternative splicing in human tissues. Genome Biol..

[23] Scott A.J., Chiang C., Hall I.M. (2021). Structural variants are a major source of gene expression differences in humans and often affect multiple nearby genes. Genome Res..

[24] Brandler W.M., Antaki D., Gujral M., Kleiber M.L., Whitney J., Maile M.S., Hong O., Chapman T.R., Tan S., Tandon P. (2018). Paternally inherited cis-regulatory structural variants are associated with autism. Science.

[25] Bartlett C.W., Hou L., Flax J.F., Hare A., Cheong S.Y., Fermano Z., Zimmerman-Bier B., Cartwright C., Azaro M.A., Buyske S. (2014). A genome scan for loci shared by autism spectrum disorder and language impairment. Am. J. Psychiatry.

[26] Evans J.L., Brown T.T., Hickok G., Small S.L. (2016). Chapter 72—Specific Language Impairment. Neurobiology of Language.

[27] NIH Developmental Language Disorder. https://www.nidcd.nih.gov/health/developmental-language-disorder.

[28] Bartlett C.W., Flax J.F., Logue M.W., Vieland V.J., Bassett A.S., Tallal P., Brzustowicz L.M. (2002). A major susceptibility locus for specific language impairment is located on 13q21. Am. J. Hum. Genet..

[29] Bartlett C.W., Flax J.F., Logue M.W., Smith B.J., Vieland V.J., Tallal P., Brzustowicz L.M. (2004). Examination of potential overlap in autism and language loci on chromosomes 2, 7, and 13 in two independent samples ascertained for specific language impairment. Hum. Hered..

[30] Bradford Y., Haines J., Hutcheson H., Gardiner M., Braun T., Sheffield V., Cassavant T., Huang W., Wang K., Vieland V. (2001). Incorporating language phenotypes strengthens evidence of linkage to autism. Am. J. Med. Genet..

[31] Bartlett C.W., Flax J.F., Fermano Z., Hare A., Hou L., Petrill S.A., Buyske S., Brzustowicz L.M. (2012). Gene x gene interaction in shared etiology of autism and specific language impairment. Biol. Psychiatry.

[32] Zhou A., Cao X., Mahaganapathy V., Azaro M., Gwin C., Wilson S., Buyske S., Bartlett C.W., Flax J.F., Brzustowicz L.M. (2023). Common genetic risk factors in ASD and ADHD co-occurring families. Hum. Genet..

[33] Li Y., Sun C., Guo Y., Qiu S., Li Y., Liu Y., Zhong W., Wang H., Cheng Y., Liu Y. (2022). DIP2C polymorphisms are implicated in susceptibility and clinical phenotypes of autism spectrum disorder. Psychiatry Res..

[34] De Rubeis S., He X., Goldberg A.P., Poultney C.S., Samocha K., Cicek A.E., Kou Y., Liu L., Fromer M., Walker S. (2014). Synaptic, transcriptional and chromatin genes disrupted in autism. Nature.

[35] Wang S., Mandell J.D., Kumar Y., Sun N., Morris M.T., Arbelaez J., Nasello C., Dong S., Duhn C., Zhao X. (2018). De Novo Sequence and Copy Number Variants Are Strongly Associated with Tourette Disorder and Implicate Cell Polarity in Pathogenesis. Cell Rep..

[36] Chaimowicz C., Ruffault P.L., Cheret C., Woehler A., Zampieri N., Fortin G., Garratt A.N., Birchmeier C. (2019). Teashirt 1 (Tshz1) is essential for the development, survival and function of hypoglossal and phrenic motor neurons in mouse. Development.

[37] Mascheretti S., Riva V., Feng B., Trezzi V., Andreola C., Giorda R., Villa M., Dionne G., Gori S., Marino C. (2020). The Mediation Role of Dynamic Multisensory Processing Using Molecular Genetic Data in Dyslexia. Brain Sci..

[38] Kato H., Kushima I., Mori D., Yoshimi A., Aleksic B., Nawa Y., Toyama M., Furuta S., Yu Y., Ishizuka K. (2020). Rare genetic variants in the gene encoding histone lysine demethylase 4C (KDM4C) and their contributions to susceptibility to schizophrenia and autism spectrum disorder. Transl. Psychiatry.

[39] Maitra S., Khandelwal N., Kootar S., Sant P., Pathak S.S., Reddy S.K.A.P., Murty U.S., Chakravarty S., Kumar A. (2020). Histone Lysine Demethylase JMJD2D/KDM4D and Family Members Mediate Effects of Chronic Social Defeat Stress on Mouse Hippocampal Neurogenesis and Mood Disorders. Brain Sci..

[40] Saez M.A., Fernandez-Rodriguez J., Moutinho C., Sanchez-Mut J.V., Gomez A., Vidal E., Petazzi P., Szczesna K., Lopez-Serra P., Lucariello M. (2016). Mutations in JMJD1C are involved in Rett syndrome and intellectual disability. Genet. Med..

[41] Jiang-Xie L.F., Liao H.M., Chen C.H., Chen Y.T., Ho S.Y., Lu D.H., Lee L.J., Liou H.H., Fu W.M., Gau S.S. (2014). Autism-associated gene Dlgap2 mutant mice demonstrate exacerbated aggressive behaviors and orbitofrontal cortex deficits. Mol. Autism.

[42] Lee J.A., Damianov A., Lin C.H., Fontes M., Parikshak N.N., Anderson E.S., Geschwind D.H., Black D.L., Martin K.C. (2016). Cytoplasmic Rbfox1 Regulates the Expression of Synaptic and Autism-Related Genes. Neuron.

[43] Lee C.T., Bendriem R.M., Kindberg A.A., Worden L.T., Williams M.P., Drgon T., Mallon B.S., Harvey B.K., Richie C.T., Hamilton R.S. (2015). Functional consequences of 17q21.31/WNT3-WNT9B amplification in hPSCs with respect to neural differentiation. Cell Rep..

[44] Eising E., Carrion-Castillo A., Vino A., Strand E.A., Jakielski K.J., Scerri T.S., Hildebrand M.S., Webster R., Ma A., Mazoyer B. (2019). A set of regulatory genes co-expressed in embryonic human brain is implicated in disrupted speech development. Mol. Psychiatry.

[45] Brunetti-Pierri N., Berg J.S., Scaglia F., Belmont J., Bacino C.A., Sahoo T., Lalani S.R., Graham B., Lee B., Shinawi M. (2008). Recurrent reciprocal 1q21.1 deletions and duplications associated with microcephaly or macrocephaly and developmental and behavioral abnormalities. Nat. Genet..

[46] Wong A., Zhou A., Cao X., Mahaganapathy V., Azaro M., Gwin C., Wilson S., Buyske S., Bartlett C.W., Flax J.F. (2022). MicroRNA and MicroRNA-Target Variants Associated with Autism Spectrum Disorder and Related Disorders. Genes.

[47] Treangen T.J., Salzberg S.L. (2011). Repetitive DNA and next-generation sequencing: Computational challenges and solutions. Nat. Rev. Genet..

[48] Pinto D., Darvishi K., Shi X., Rajan D., Rigler D., Fitzgerald T., Lionel A.C., Thiruvahindrapuram B., Macdonald J.R., Mills R. (2011). Comprehensive assessment of array-based platforms and calling algorithms for detection of copy number variants. Nat. Biotechnol..

[49] Cukier H.N., Skaar D.A., Rayner-Evans M.Y., Konidari I., Whitehead P.L., Jaworski J.M., Cuccaro M.L., Pericak-Vance M.A., Gilbert J.R. (2009). Identification of chromosome 7 inversion breakpoints in an autistic family narrows candidate region for autism susceptibility. Autism Res..

[50] Tabet A.C., Verloes A., Pilorge M., Delaby E., Delorme R., Nygren G., Devillard F., Gerard M., Passemard S., Heron D. (2015). Complex nature of apparently balanced chromosomal rearrangements in patients with autism spectrum disorder. Mol. Autism.

[51] Zhou A., Lin T., Xing J. (2019). Evaluating nanopore sequencing data processing pipelines for structural variation identification. Genome Biol..

[52] Guo Y., He J., Zhao S., Wu H., Zhong X., Sheng Q., Samuels D.C., Shyr Y., Long J. (2014). Illumina human exome genotyping array clustering and quality control. Nat. Protoc..

[53] Axiom™ Analysis Suite v4.0.1 User Guide.

[54] Purcell S., Neale B., Todd-Brown K., Thomas L., Ferreira M.A., Bender D., Maller J., Sklar P., de Bakker P.I., Daly M.J. (2007). PLINK: A tool set for whole-genome association and population-based linkage analyses. Am. J. Hum. Genet..

[55] Wang K., Li M., Hadley D., Liu R., Glessner J., Grant S.F., Hakonarson H., Bucan M. (2007). PennCNV: An integrated hidden Markov model designed for high-resolution copy number variation detection in whole-genome SNP genotyping data. Genome Res..

[56] Colella S., Yau C., Taylor J.M., Mirza G., Butler H., Clouston P., Bassett A.S., Seller A., Holmes C.C., Ragoussis J. (2007). QuantiSNP: An Objective Bayes Hidden-Markov Model to detect and accurately map copy number variation using SNP genotyping data. Nucleic Acids Res..

[57] Geoffroy V., Herenger Y., Kress A., Stoetzel C., Piton A., Dollfus H., Muller J. (2018). AnnotSV: An integrated tool for structural variations annotation. Bioinformatics.

[58] Quinlan A.R., Hall I.M. (2010). BEDTools: A flexible suite of utilities for comparing genomic features. Bioinformatics.

[59] Paila U., Chapman B.A., Kirchner R., Quinlan A.R. (2013). GEMINI: Integrative Exploration of Genetic Variation and Genome Annotations. PLoS Comput. Biol..

[60] Aguet F., Brown A.A., Castel S.E., Davis J.R., He Y., Jo B., Mohammadi P., Park Y., Parsana P., Segrè A.V. (2017). Genetic effects on gene expression across human tissues. Nature.

[61] Aguet F., Barbeira A.N., Bonazzola R., Brown A., Castel S.E., Jo B., Kasela S., Kim-Hellmuth S., Liang Y., Oliva M. (2019). The GTEx Consortium atlas of genetic regulatory effects across human tissues. bioRxiv.

[62] Miller J.A., Ding S.L., Sunkin S.M., Smith K.A., Ng L., Szafer A., Ebbert A., Riley Z.L., Royall J.J., Aiona K. (2014). Transcriptional landscape of the prenatal human brain. Nature.

[63] Lindsay S.J., Xu Y.B., Lisgo S.N., Harkin L.F., Copp A.J., Gerrelli D., Clowry G.J., Talbot A., Keogh M.J., Coxhead J. (2016). HDBR Expression: A Unique Resource for Global and Individual Gene Expression Studies during Early Human Brain Development. Front. Neuroanat..

[64] Mohiyuddin M., Mu J.C., Li J., Bani Asadi N., Gerstein M.B., Abyzov A., Wong W.H., Lam H.Y. (2015). MetaSV: An accurate and integrative structural-variant caller for next generation sequencing. Bioinformatics.

[65] Gardner E.J., Lam V.K., Harris D.N., Chuang N.T., Scott E.C., Pittard W.S., Mills R.E., Genomes Project C., Devine S.E. (2017). The Mobile Element Locator Tool (MELT): Population-scale mobile element discovery and biology. Genome Res..

[66] Li H. (2011). A statistical framework for SNP calling, mutation discovery, association mapping and population genetical parameter estimation from sequencing data. Bioinformatics.

[67] Zhao H., Sun Z., Wang J., Huang H., Kocher J.P., Wang L. (2014). CrossMap: A versatile tool for coordinate conversion between genome assemblies. Bioinformatics.

[68] Jeffares D.C., Jolly C., Hoti M., Speed D., Shaw L., Rallis C., Balloux F., Dessimoz C., Bahler J., Sedlazeck F.J. (2017). Transient structural variations have strong effects on quantitative traits and reproductive isolation in fission yeast. Nat. Commun..

[69] Collins R.L., Brand H., Karczewski K.J., Zhao X., Alfoldi J., Francioli L.C., Khera A.V., Lowther C., Gauthier L.D., Wang H. (2020). A structural variation reference for medical and population genetics. Nature.

[70] Cao X., Zhang Y., Abdulkadir M., Deng L., Fernandez T.V., Garcia-Delgar B., Hagstrom J., Hoekstra P.J., King R.A., Koesterich J. (2021). Whole-exome sequencing identifies genes associated with Tourette’s disorder in multiplex families. Mol. Psychiatry.

[71] Sharo A.G., Hu Z., Sunyaev S.R., Brenner S.E. (2022). StrVCTVRE: A supervised learning method to predict the pathogenicity of human genome structural variants. Am. J. Hum. Genet..

[72] Danis D., Jacobsen J.O.B., Balachandran P., Zhu Q., Yilmaz F., Reese J., Haimel M., Lyon G.J., Helbig I., Mungall C.J. (2022). SvAnna: Efficient and accurate pathogenicity prediction of coding and regulatory structural variants in long-read genome sequencing. Genome Med..

[73] Herwig R., Hardt C., Lienhard M., Kamburov A. (2016). Analyzing and interpreting genome data at the network level with ConsensusPathDB. Nat. Protoc..

[74] Hagberg A., Swart P., S Chult D. (2008). Exploring Network Structure, Dynamics, and Function Using Networkx.

[75] Szklarczyk D., Morris J.H., Cook H., Kuhn M., Wyder S., Simonovic M., Santos A., Doncheva N.T., Roth A., Bork P. (2017). The STRING database in 2017: Quality-controlled protein-protein association networks, made broadly accessible. Nucleic Acids Res.

[76] Greene C.S., Krishnan A., Wong A.K., Ricciotti E., Zelaya R.A., Himmelstein D.S., Zhang R., Hartmann B.M., Zaslavsky E., Sealfon S.C. (2015). Understanding multicellular function and disease with human tissue-specific networks. Nat. Genet..

[77] Wong A.K., Krishnan A., Troyanskaya O.G. (2018). GIANT 2.0: Genome-scale integrated analysis of gene networks in tissues. Nucleic Acids Res..

