# Supplementary material for: Structural Variations Contribute to the Genetic Etiology of Autism Spectrum Disorder and Language Impairments
Source: Int J Mol Sci. 2023 Aug 26;24(17):13248. doi: 10.3390/ijms241713248 (PMC10487745; doi:10.3390/ijms241713248)
Supplement: Supplementary file 1 [file ijms-24-13248-s001.zip › SuppFigsTables_Revision_2023_08_23/SupplementalFigures.pdf]

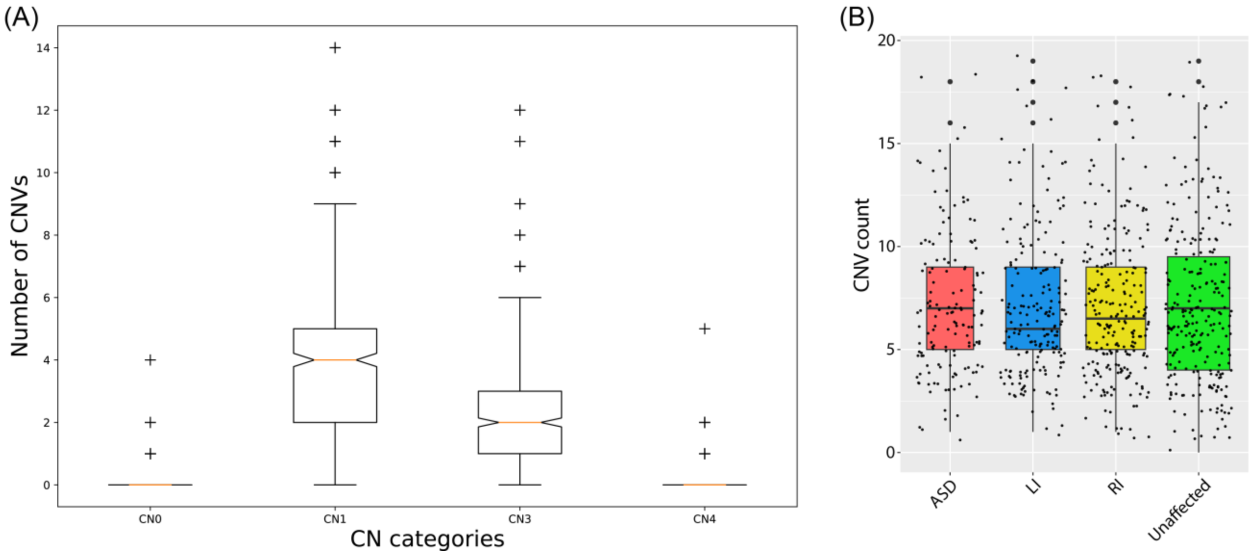

Figure S1. (A) CNV copy number distribution in each individual. CN: copy number. For example, CN0 represents copy number 0 (i.e., homozygous deletion). Normal diploid genome has a copy number 2 (CN2, not shown). **(B) CNV burden in ASD, LI, and RI patients and unaffected family members.** Each dot is the number of CNVs in each individual. The box-whisker plot shows the median and quantiles for each group.

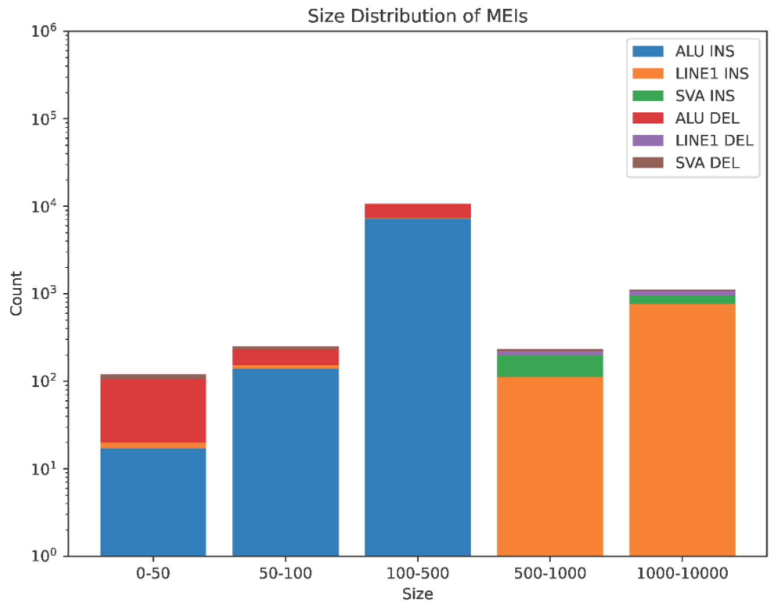

Figure S2. Size distribution of MEIs. INS: insertions. DEL: deletions (*i.e.*, MEIs present in the reference genome but not in the sequenced individual, see Methods for detail).
